# The design of a multi-arm multi-stage (MAMS) phase III randomised controlled trial comparing alternative regimens for escalating (COMPARE) treatment of intermediate and high-risk oropharyngeal cancer with reflections on the complications of introducing a new experimental ARM

**DOI:** 10.1186/1745-6215-16-S2-O16

**Published:** 2015-11-16

**Authors:** Piers Gaunt, Hisham Mehanna, Christina Yap

**Affiliations:** 1 Cancer Research Clinical Trials Unit, University of Birmingham, Birmingham, UK; 2 Institute of Head and Neck Studies and Education, University of Birmingham, Birmingham, UK

## 

CompARE is a pragmatic multicentre open-label phase III randomised controlled trial aiming to determine if intensification of treatment in intermediate and high risk oropharyngeal cancer (OPC) patients improves the definitive primary outcome measure of overall survival time. The trial evaluates three experimental arms separately against one control arm using a MAMS design, with three interim assessments of disease-free survival time. Experimental arms will be discontinued if they fail to meet the interim assessment criteria. The timing of these assessments is driven by the number of control events, with the study engineered so these occur approximately annually. The design characteristics will be presented.

A potential additional experimental arm for treatment of OPC was proposed during CompARE initiation, and could be introduced into the trial after one year if approved. The straightforward implication is an increase in the number of patients required to recruit per year or an increase in trial duration. However in a complex MAMS design, a balance of multiple factors such as a feasible sample size, trial duration and appropriate number and timing of interim assessments with appropriate statistical error rates, have to be carefully considered.

Using the nstage and artpep programs in Stata, a review of the operating characteristics of both the original design and expanded design with the new experimental arm was undertaken. The recruitment and statistical implications of the addition of the new experimental arm and an evaluation of the study duration to changes in recruitment predictions will be presented.

